# On the Potential Self-Amplification of Aneurysms Due to Tissue Degradation and Blood Flow Revealed From FSI Simulations

**DOI:** 10.3389/fphys.2021.785780

**Published:** 2021-12-10

**Authors:** Haifeng Wang, Daniel Balzani, Vijay Vedula, Klemens Uhlmann, Fathollah Varnik

**Affiliations:** ^1^Theory and Simulation of Complex Fluids, Department of Scale-Bridging Thermodynamic and Kinetic Simulation, Interdisciplinary Center for Advanced Materials Simulation (ICAMS), Ruhr-Universität Bochum, Bochum, Germany; ^2^Department of Civil and Environmental Engineering, Chair of Continuum Mechanics, Ruhr-Universität Bochum, Bochum, Germany; ^3^Department of Mechanical Engineering, Columbia University in the City of New York, New York, NY, United States

**Keywords:** aneurysms, tissue degradation, fluid-structure interaction (FSI), hemodynamics, wall shear stress (WSS), oscillatory shear index (OSI)

## Abstract

Tissue degradation plays a crucial role in the formation and rupture of aneurysms. Using numerical computer simulations, we study the combined effects of blood flow and tissue degradation on intra-aneurysm hemodynamics. Our computational analysis reveals that the degradation-induced changes of the time-averaged wall shear stress (TAWSS) and oscillatory shear index (OSI) within the aneurysm dome are inversely correlated. Importantly, their correlation is enhanced in the process of tissue degradation. Regions with a low TAWSS and a high OSI experience still lower TAWSS and higher OSI during degradation. Furthermore, we observed that degradation leads to an increase of the endothelial cell activation potential index, in particular, at places experiencing low wall shear stress. These findings are robust and occur for different geometries, degradation intensities, heart rates and pressures. We interpret these findings in the context of recent literature and argue that the degradation-induced hemodynamic changes may lead to a self-amplification of the flow-induced progressive damage of the aneurysmal wall.

## 1. Introduction

Both microscopic degradation in vascular tissues and hemodynamic (fluid-dynamic) forces play a crucial role in the initiation, growth and focal rupture of aneurysms (Sforza et al., 2009; Salman et al., 2019; Lipp et al., 2020; Wu et al., 2020). Aneurysms are vascular diseases characterized by excessive tissue degradation and chronic inflammation (Frösen, 2014). There are relations among aneurysmal geometry, intra-aneurysmal hemodynamics (flow), and aneurysm pathobiology (Meng et al., 2014): Geometry instantaneously alters flow conditions (short-term effect) (Wang et al., 2020, 2021a); abnormal-flow-induced hemodynamic-biomechanical triggers are transduced into biological signals and lead to the degradation, growth and/or remodeling of aneurysms via pathobiology (Meng et al., 2014); the interplay between the local flow environment and aneurysm pathobiology dominates the growth and geometric changes of the aneurysm (long-term effect) (Tarbell et al., 2014). Within an aneurysm wall, constructive (eutrophic) changes (cell proliferation and extracellular matrix production) and destructive (degradative) changes (cell death and extracellular matrix degradation) are ongoing concurrently (Frösen et al., 2012; Frösen, 2014; Meng et al., 2014). Wall shear stress (WSS), defined as the tangential stress component induced by the flowing blood and acting on endothelial cells, regulates the near-wall transport of chemicals and proteins (Kadirvel et al., 2007; Nixon et al., 2010; Meng et al., 2014). WSS, however, is usually calculated for a stationary/instantaneous blood flow and does not, per se, consider the pulsatility within a cardiac cycle. In order to better elucidate the mechanistic link between blood flow and vascular diseases (in particular, aneurysms), a number of WSS-related metrics have been proposed. Among these, the time-averaged wall shear stress (TAWSS) (He and Ku, 1996) and oscillatory shear index (OSI) (Ku et al., 1985) are the two most common candidates that are important for aneurysms' progression and rupture. TAWSS measures the average *magnitude* of WSS within a full cardiac cycle. OSI indicates the change in the *direction* along which WSS is acting on the vascular tissue. Abnormal WSS is a major cause of the imbalance between the constructive and destructive processes (Meng et al., 2014) and leads to vascular degradation and inflammation by activating inflammatory markers of endothelial cells (Franck et al., 2013; Meng et al., 2014), thereby causing the breakdown of the internal elastic lamina and loss of structural strength within the vessel wall (Kataoka et al., 1999). An aneurysm can grow and even rupture with continuous vascular injury, inflammation, and prolonged activation (Fisher and Demel, 2019).

On the one hand, although mechanisms underlying the effects of hemodynamic forces on aneurysm pathogenesis remain unclear, there is growing evidence that hemodynamic factors (in particular, TAWSS and OSI) act as crucial contributors to the progression and rupture of aneurysms. Low TAWSS and high OSI are commonly used risk factors for a rupture-prone phenotype (Xiang et al., 2011; Meng et al., 2014; Zhang et al., 2016; Liu et al., 2019a). Previous *in vitro* (Davies et al., 1984; Dai et al., 2004) and numerical (Sáez et al., 2015) studies have indicated that the remodeling of endothelial cells is dependent on the combined effects of TAWSS and OSI. Liu et al. (2019a) have recently suggested that low WSS and high OSI shall be considered as independent hemodynamic-morphological risk factors and proposed to use them as predictors for intra-operative aneurysm rupture. Low TAWSS and high OSI have also been shown to be significantly associated with thrombus formation in aneurysms (Les et al., 2010; Kelsey et al., 2017). In addition, Cebral et al. (2019) have argued that high WSS and low OSI, prevalent in the flow impingement region, may be also associated with the degradation and local thinning of aneurysmal walls. Both the TAWSS and OSI affect the endothelial mechanobiology locally. To localize regions exposed to both low TAWSS and high OSI, the so-called endothelial cell activation potential (ECAP) is used (Di Achille et al., 2014; Zambrano et al., 2016; Kelsey et al., 2017; Ong et al., 2019). It has been shown that low TAWSS, high OSI ang high ECAP correlate with regions of thrombus development in aneurysms (Zambrano et al., 2016; Kelsey et al., 2017; Ong et al., 2019). The effects of geometry on intra-aneurysmal hemodynamics have also been extensively studied (Hassan et al., 2005; Cebral et al., 2007; Baharoglu et al., 2010; Kawaguchi et al., 2012; Wang et al., 2020).

On the other hand, degradation-induced changes in mechanical properties of an aneurysmal wall may have an influence on hemodynamics inside the aneurysm, which is poorly studied though. Recently, we have developed a novel computational framework by combining a tissue degradation model and a finite element-based fluid-structure interaction (FSI) solver (Wang et al., 2021b). Using this model, we have shown that TAWSS increases near the flow-impingement region of idealized aneurysms and decreases away from it in the process of degradation (Wang et al., 2021b).

While several longitudinal studies (Arzani et al., 2014; Zambrano et al., 2016) have been conducted to understand the role of TAWSS and OSI in thrombus deposition in aneurysms, it remains unclear how degradation may affect the oscillatory shear index and its potential connection to TAWSS, and the endothelial cell activation potential. The present study aims at investigating this topic. To the best of our knowledge, this paper presents the first preliminary results uncovering qualitative trends and correlations of biomechanically important metrics upon degradation. Potential consequences of the central finding are discussed in the context of recent literature.

## 2. Materials and Methods

### 2.1. Numerical Model

As mentioned above, in this work, we use a recently developed FSI framework to account for the interaction between blood flow and aneurysmal walls, and at the same time to capture the degradation of vascular tissues (Wang et al., 2021b). In this computational framework, a tissue degradation model (Balzani et al., 2012; Anttila et al., 2019) is combined with the open-source software, SimVascular/svFSI (2021) which is finite-element-method-based and uses an arbitrary Lagrangian-Eulerian formulation of Navier-Stokes equations to model incompressible Newtonian fluid (blood) flows on moving domains (Vedula et al., 2017). The degradation model employed here has been validated in previous studies (Balzani et al., 2012; Anttila et al., 2019) and proved to reproduce the experimental cyclic responses of different types of arteries. The current model is able to account for stress softening (Balzani et al., 2012; Anttila et al., 2019; Wang et al., 2021b), a phenomenon commonly observed in biological tissues. The present study is qualitative research and a first step toward a more realistic model.

**Figure 1:**
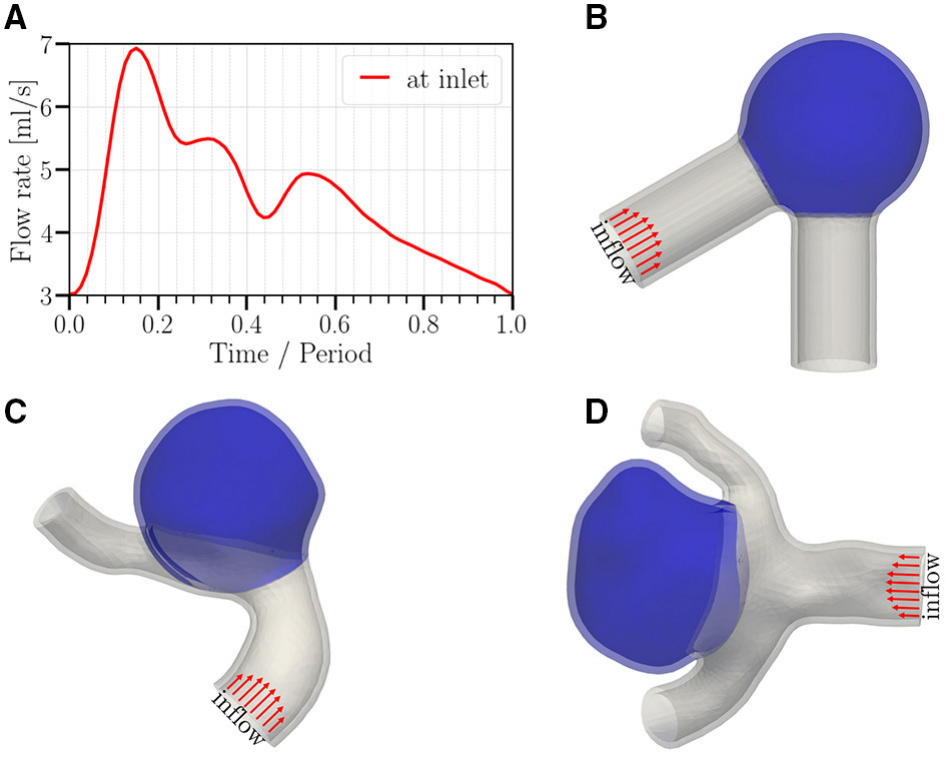
**(A)** Temporal variation of the imposed flow rate at the inlet (period *T* = 0.6 s or 0.8 s, depending on the studied case). **(B–D)** Geometries studied in this work; **(B)** a canonical aneurysm model based on data in (Usmani and Muralidhar, 2018; Chassagne et al., 2021); **(C,D)** patient-specific cerebral aneurysm models based on the cases C0034 and C0020 in the open-source *Aneurisk* dataset repository (Aneurisk-Team, 2012), respectively. In order to keep the computational cost in a reasonable range, we select a short representative section from each full image available in the *Aneurisk* repository. All meshes use quadratic tetrahedrons having a mean effective spatial resolution of 0.07 mm. Arrows serve to highlight the parabolic velocity profile imposed at the inlet. The tissue degradation model is applied only on the aneurysmal region (colored in blue); the remaining of the vessel (gray) is modeled as Neo-Hookean.

For flow boundary conditions, we prescribe a pulsatile flow (Figure 1A) at the inlet with a parabolic velocity profile, together with a three-element Windkessel-type boundary condition at each outlet. The Windkessel parameters are tuned to match the systolic, diastolic and mean blood pressures. At each cross-sectional end of the structural domain, homogeneous Dirichlet boundary conditions are applied to anchor its location. The fluid and wall domains share a contact surface. Through each contact surface point, the flow velocity is projected onto the wall. Indeed, we impose the non-slip condition on the surface regardless of the thickness of the wall.

### 2.2. Study Cases

Three geometries are employed in this study (Figure 1). For the idealized aneurysm (Figure 1B), the inner diameter of the parent vessel is 0.41 cm (Chnafa et al., 2017) with an angle of 60° between the two vessel parts. The inner diameter of the spherical cap (aneurysm) is 1.0 cm. The mean diameter of the parent vessel in Figures 1C,D is approximately identical and equal to 0.4 cm. The average diameter of the aneurysm dome in Figures 1C,D is 1.2 and 0.8 cm, respectively. The aspect ratio (perpendicular height to neck diameter) of the aneurysm sac in Figures 1C,D is approximately 1.0 and 1.2, respectively.

**Figure 2:**
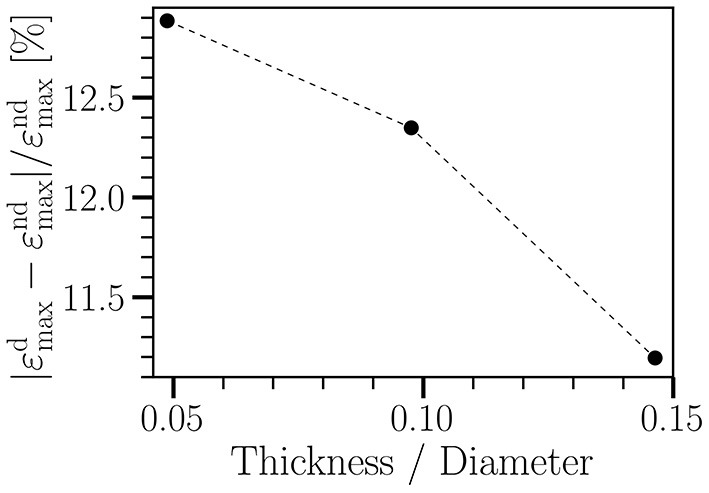
Relative change in radial strain of a straight tube between non-degraded (ε^nd^) and degraded (ε^d^) cases vs. wall thickness of the tube. The tube diameter is 0.41 cm. The wall thickness varies from 0.02 cm to 0.06 cm. The imposed cyclic load ranges from 8.7 kPa to 20 kPa, having the same waveform as shown in Figure 1A. Each simulation runs for five cycles. *T* = 0.8 s; γ_∞_ = 18 kPa. Other material parameters are given in Table 1.

Following the literature, the wall thickness has been chosen to be 0.04cm (Isaksen et al., 2008; Wang et al., 2021b). As shown in Figure 2, a variation of wall thickness by a factor of three (from 0.02 to 0.06 cm) leads to less than 15% change in degradation-induced effect on strain. Other quantities behave similarly (not shown). Therefore, for simplicity, we assume a spatially homogeneous wall thickness.

Unless otherwise stated, material parameters given in Table 1 are used in this study. The fluid density and viscosity are 1.055 g cm^−3^ and 0.04 g cm^−1^ s^−1^, respectively (Isaksen et al., 2008).

**Table 1:** Material parameters for the aneurysm region (colored in blue in Figures 1B–D) used in this study [based on data shown in Balzani et al. (2012)].

***c*_1_ [kPa]**	***ϵ*_1_ [kPa]**	***ϵ*_2_ [-]**	***α*_1_ [kPa]**	***α*_2_ [-]**	***κ* [-]**	***β*_f_ [**°**]**	***D*_∞_ [kPa]**	**γ_∞_ [kPa]**	**β_s_ [-]**
9.02	499.8	2.4	1,400	2.2	1e-8	39.87	0.96	11	0.06

*For the parent and branch vessels outside an aneurysm (gray regions shown in Figures 1B–D), elastic modulus and Poisson's ratio are chosen as 1,000 kPa (Khamdaeng et al., 2012) and 0.49, respectively. See Supplementary Material for more details on how the material parameters enter the tissue degradation model*.

The pressure levels are controlled by varying the three-element Windkessel parameters (i.e., distal resistance *R*_d_, proximal resistance *R*_p_, and capacitance *C*). For a bifurcation artery, the total resistance, *R*_tot_, at each outlet is calculated assuming the power law relationship between the blood flow rate and the internal vessel diameter (Chnafa et al., 2017). The distal and proximal resistance at each outlet is given by *R*_d_ = *k*_d_*R*_tot_, and *R*_p_ = (1−*k*_d_)*R*_tot_, where the factor *k*_d_ defines the ratio of distal to total resistance and is fixed for all outlets to *k*_d_ = 0.9 (Kim et al., 2010). *R*_d_ is often chosen to be higher than *R*_p_ as the most resistance occurs in the downstream vascular system. The capacitance *C* controls the amplitude of the pressure waveform; a low capacitance leads to a high pressure amplitude.

In order to gain a qualitative understanding of the effects arising from tissue degradation, we start with an idealized aneurysm model shown in Figure 1B with pressure ranging from 90 mmHg (diastole) to 160 mmHg (systole) and a heart rate of 100 bpm (*T* = 0.6 s). The total resistance and capacitance at each branch are *R*_tot_ = 7.3·10^4^ g cm^−4^ s^−1^ and *C* = 10^−6^ cm^4^ s^2^ g^−1^, respectively. We then investigate the influence of different parameters as follows:

1. Morphology-related effects: Here we perform simulations using the same parameters as described above for two patient-specific aneurysm geometries (Figures 1C,D).
2. Effect of degradation intensity: In this case we change the value of the damage parameter γ_∞_ from 11 to 18 kPa ; note that γ_∞_ = 18kPa corresponds to a lower damage intensity than the case with γ_∞_ = 11kPa (see Supplementary Material and Wang et al., 2021b).
3. Influence of heart rate (flow frequency): Here the heart rate is changed from 100 bpm (*T* = 0.6 s) to 75 bpm (*T* = 0.8 s) for the two patient-specific geometries.
4. Pressure (tension)-dependence: Here we impose a different blood pressure ranging from 70 mmHg (diastole) to 140 mmHg (systole), again for the two patient-specific aneurysms.

In order to highlight effects arising from the degradation of blood vessel tissue, all the above simulations are performed twice: once with and once without material degradation.

All meshes use quadratic tetrahedrons (Balzani et al., 2016; Wang et al., 2021b). For each case investigated in the present study, the fluid and solid domains consist of approximately 93,000 nodes/67,000 tetrahedral elements. The discrete time step is set to 10^−4^ s; for a cycle with a duration of 0.8 s, the temporal resolution is 8,000 time-steps per cardiac cycle. As to the computation time, each simulation with a typical duration of five cardiac cycles takes roughly 10 days using 38 cores on a multi-core workstation (Intel(R) Xeon(R) Gold 6148, 2.40 GHz).

### 2.3. Metrics for Analysis

As motivated in the Introduction, time-averaged wall shear stress and oscillatory shear index play a pivotal role in mechanobiological development and focal rupture of aneurysms.

The TAWSS at an arbitrary position ***x*** is simply the average magnitude of the wall shear stress vector **τ***_w_* over one cardiac cycle of duration *T* at that point (He and Ku, 1996),


(1) $$\begin{array}{r} \text{TAWSS}\left(x\right)=\frac{1}{T}\int_{0}^{T}|\tau_{\text{w}}\left(x,t\right)|\;\text{d}t. \end{array}$$

The oscillatory shear index quantifies the change in the orientation of the wall shear stress vector during a cardiac cycle and is calculated as (Ku et al., 1985),


(2) $$\begin{array}{r} \text{OSI}\left(x\right)=0.5\left(1-\frac{\left|\frac{1}{T}\int_{0}^{T}\tau_{\text{w}}\left(x,t\right)\;\text{d}t\right|}{\text{TAWSS}\left(x\right)}\right). \end{array}$$

The value of OSI ranges from 0 in a uni-directional flow to 0.5 in a reversing flow with a 180° change in the direction of the shear force acting on the tissue surface.

Another interesting metric is the so-called endothelial cell activation potential, ECAP, which combines TAWSS and OSI as


(3) $$\begin{array}{r} \text{ECAP}\left(x\right)=\frac{\text{OSI}\left(x\right)}{\text{TAWSS}\left(x\right)}. \end{array}$$

The ECAP is usually used to characterize the degree of ‘thrombotic susceptibility' of arterial walls (Di Achille et al., 2014). A large value of the ECAP signals low TAWSS together with high OSI and vise versa. All the above-mentioned hemodynamic quantities are used to quantify the flow environment and flow-induced shear stress experienced by endothelium.

To quantify the effects of tissue degradation on intra-aneurysm hemodynamics, we analyze the relative changes in these metrics upon degradation. For this purpose, given a time-averaged quantity *f*(***x***) at point ***x***, the relative percentage change in *f*(***x***) between the degraded (*f*^d^) and non-degraded (*f*^nd^) cases is defined via


(4) $$\begin{array}{r} \begin{array}{c} \Delta f\left(x\right)=\frac{f^{\text{d}}\left(x\right)-f^{\text{nd}}\left(x\right)}{f^{\text{nd}}\left(x\right)}\times 100. \end{array} \end{array}$$

## 3. Results

In this section, we investigate the effect of tissue degradation on two commonly used intra-aneurysmal hemodynamic quantities, TAWSS and OSI, and ECAP. Since the fluid is at rest at the beginning of our simulations, we wait for two full cardiac cycles before gathering the data on WSS and OSI. This way, we focus on cyclic dynamics, which regularly repeats itself.

**Figure 3:**
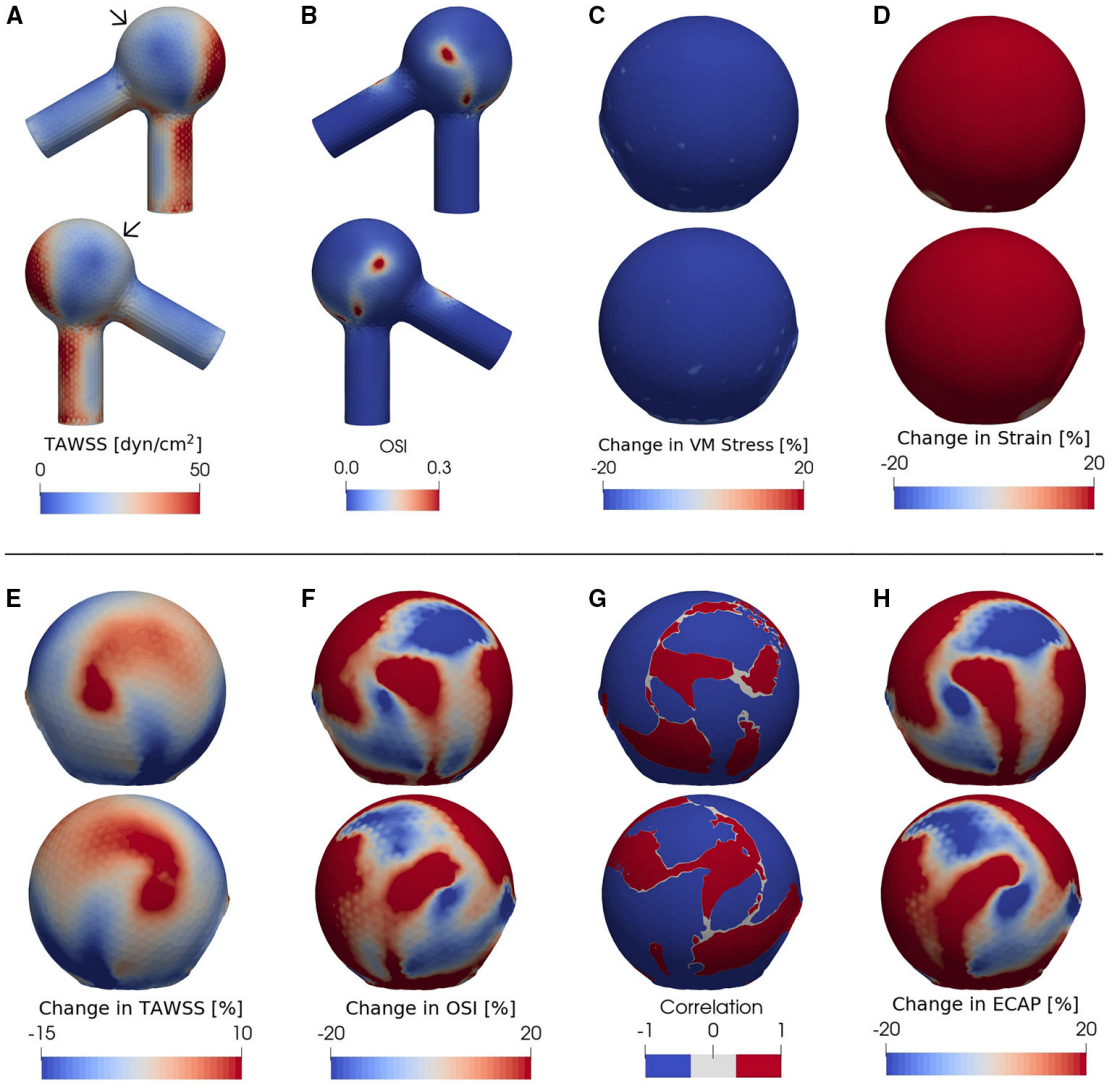
**(A)** TAWSS and **(B)** OSI fields in the presence of degradation using an idealized aneurysm model. Relative percentage change (Equation 4) of **(C)** Von Mises stress, **(D)** strain magnitude, **(E)** TAWSS and **(F)** OSI between non-degraded and degraded cases; the blue (red) color indicates a decrease (increase) of each metric upon tissue degradation. Panel **(G)** illustrates the correlation function between the data shown in the panels **(E,F)**, calculated via Equation (5) and digitized to –1 or 1 for all data points with a magnitude >5%. The region with blue color corresponds to an inverse effect of degradation on TAWSS as opposed to OSI: In places where TAWSS decreases due to degradation, OSI increases and vice versa. A positive correlation, on the other hand, is represented by a red color in **(G)**. **(H)** shows the relative change of ECAP upon degradation. In all cases, the pressure ranges from 90 (diastole) to 160 mmHg (systole) and *T* = 0.6 s. For each case investigated, the locations of low-WSS (indicated by arrows) remain nearly intact during a full cardiac cycle. In order to better visualize the data, the second (fourth) row shows the same simulated data as in the first (third) row but after rotating the aneurysm dome by an angle of π around the polar axis. In **(C–H)** we show only the aneurysm region since the degradation model is applied only on the aneurysm domain.

In order to quantify the effects of tissue degradation on TAWSS, OSI and ECAP, we use Equation (4) and determine the tissue-degradation-induced relative changes of these hemodynamic quantities (Figure 3). Using this approach, a decrease or increase of TAWSS, OSI, and ECAP upon degradation shows itself in a negative or positive value and will be made visible by blue or red color, respectively.

As revealed by our simulations, degradation effects seem to be nearly homogeneous on wall stress (Figure 3C) and strain magnitude (Figure 3D) [in line with our previous study (Wang et al., 2021b)] but spatially heterogeneous on TAWSS (Figure 3E), OSI (Figure 3F) and ECAP (Figure 3H). Importantly, the degradation process appears to bring locally opposite influences on the TAWSS and OSI; a region with a decrease in TAWSS shows an increase in OSI (and thus an increase in ECAP as visualized by red color in Figure 3H), and vice versa.

To further highlight the enhancement of the inverse correlation between TAWSS and OSI in the progress of degradation, we quantify their correlation via


(5) $$\begin{array}{r} C_{\Delta \text{TAWSS}\cdot \Delta \text{OSI}}=\frac{\text{TAWS}{\text{S}}^{\text{d}}-\text{TAWS}{\text{S}}^{\text{nd}}}{\text{TAWS}{\text{S}}^{\text{nd}}}\\ \;\times \frac{\text{OS}{\text{I}}^{\text{d}}-\text{OS}{\text{I}}^{\text{nd}}}{\text{OS}{\text{I}}^{\text{nd}}}\times 100. \end{array}$$

The degradation-induced local enhancement of the inverse correlation between TAWSS and OSI is indicated by negative *C*_ΔTAWSS·ΔOSI_ and is visualized by blue color in Figure 3G. The main advantage of introducing this quantity is that its negative value indicates opposite changes of TAWSS and OSI during degradation. When combined with the information contained in ECAP, one can then uniquely distinguish which of the two quantities has increased and which one has decreased. However, if TAWSS and OSI change in a similar manner (both increase or both decrease, leading to a positive correlation), ECAP will raise if (i) the percentage increase in OSI is larger than that of TAWSS but also if (ii) percentage decrease of OSI is lower than that of TAWSS. Similarly, two possibilities exist for a decrease of ECAP in the case of a positive correlation value.

In view of possible clinical consequences of an inverse correlation between TAWSS and OSI, we have examined the robustness of the observed behavior and have performed two new sets of simulations using patient-specific aneurysm geometries shown in Figures 1C,D. The data for these geometries are obtained from the *Aneurisk* repository (Aneurisk-Team, 2012).

**Figure 4:**
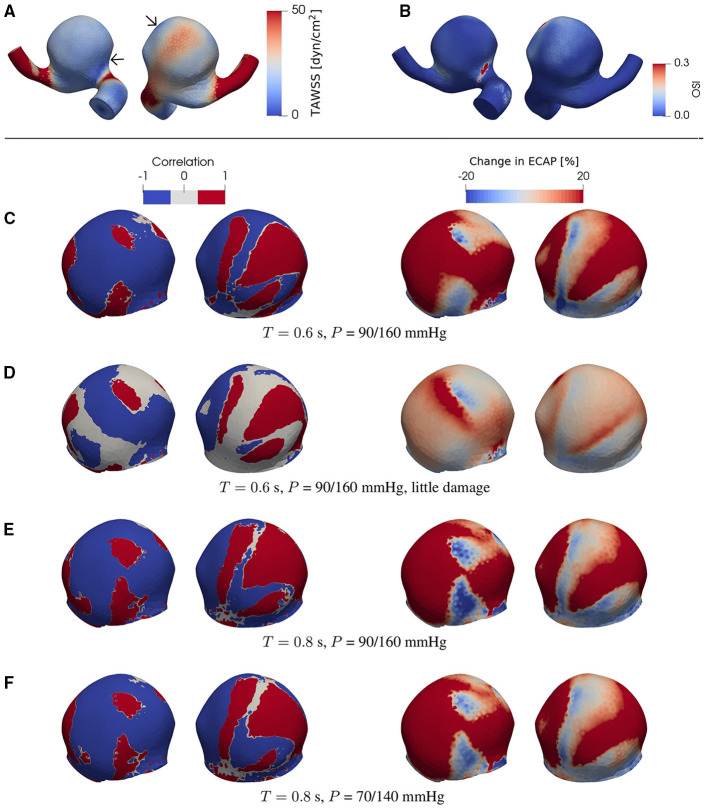
**(A)** TAWSS and **(B)** OSI field in the presence of degradation using a patient-specific aneurysm geometry [C0034 in *Aneurisk* dataset repository (Aneurisk-Team, 2012); Figure 1C]. **(C–F)** Product of degradation-induced relative changes in TAWSS and OSI (two left-hand columns; Equation 5) and the relative change in ECAP (two right-hand columns; Equation 4) under different damage intensities and flow conditions. The damage intensity in **(C)** is larger than that in **(D)**. **(C,E)** differ in only the heart rate (or duration of cardiac cycle *T*). Compared to **(E)**, the pressure *P* in **(F)** is lower. In **(C–F)**, all negative and positive products (threshold = ∓5%) of the relative changes in TAWSS and OSI are set to –1 and 1, respectively. As indicated by comparisons between **(A)** and **(C–F)**, the degradation-enhanced inverse correlation between TAWSS and OSI remains intact particularly at regions with low WSS. For each case investigated, the locations of low-WSS (indicated by arrows) remain intact during a full cardiac cycle. The second (fourth) column shows the same simulated data as in the first (third) column but after rotating the aneurysm dome by an angle of π around the polar axis. In **(C–F)** we show only the aneurysm region since the degradation model is applied only on the aneurysm domain.

**Figure 5:**
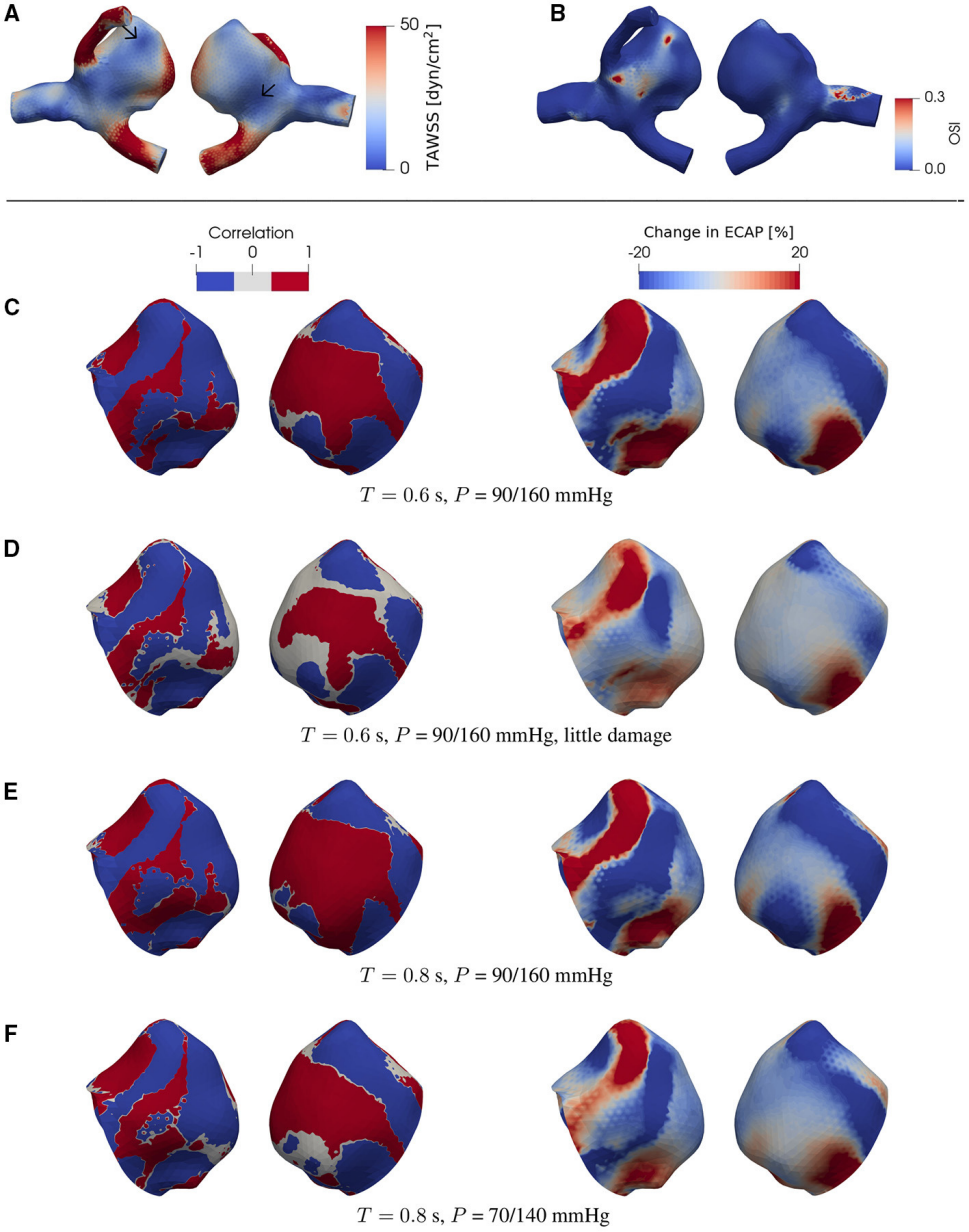
The same type of data as in Figure 4 but now for a different patient-specific aneurysm [C0020 in *Aneurisk* dataset repository (Aneurisk-Team, 2012); see as Figure 1D]. For each case investigated, the locations of low-WSS (indicated by arrows) remain intact during a full cardiac cycle.

We find that, at certain areas of each aneurysm sac investigated in this study, TAWSS and OSI are oppositely affected by degradation. This feature is stronger at places with low WSS (compare Figures 3A,G,H, 4A,C–F, 5A,C–F). In this study, a threshold value of 25 dyn/cm^2^ is used to define the low and high TAWSS, also in agreement with reported values in the literature (Mendieta et al., 2020; Morbiducci et al., 2020; Tian et al., 2021). High OSI is considered to be larger than 0.15 (Xu et al., 2020).

As will be discussed in more detail below, this feature is robust and is confirmed in the case of both ideal and patient-specific geometries, and different degradation intensities, heart rates, and blood pressures.

## 4. Discussion

Although mechanisms in aneurysm pathogenesis have not yet been fully elucidated, there is growing evidence that hemodynamic factors and tissue degradation act as crucial contributors to the progression of aneurysms (Kadirvel et al., 2007; Sforza et al., 2009; Nixon et al., 2010; Meng et al., 2014; Cebral et al., 2019; Salman et al., 2019). Abnormal flow-induced WSS often causes disruption and dysfunction of endothelial cells (Sorokin et al., 2020), initiating an aneurysm (Meng et al., 2014). Subsequently, a vascular wall may degrade due to inflammation-triggered biochemical reactions such as apoptosis and migration of smooth muscle cells, infiltration of inflammatory cells, and secretion of various cytokines (Pentimalli et al., 2004; Kwak et al., 2014; Meng et al., 2014; Liu et al., 2019b). Chronic hemodynamic stress and inflammation induce the structure change or damage in the endothelial cells and lead to the progression and eventual rupture of aneurysms (Frösen et al., 2012; Signorelli et al., 2018). Time-averaged wall shear stress and oscillatory shear index are commonly used to quantify changes in magnitude and direction of WSS.

Using computer simulations, we studied the effects of tissue degradation on these two hemodynamic stimuli, TAWSS and OSI, for the case of aneurysm geometry. The results obtained within this work revealed a strong heterogeneity in the effects arising from degradation on TAWSS and OSI.

Importantly, the degradation-induced variations of these two hemodynamic factors turned out to be opposite to each other. In places with decreased TAWSS due to tissue degradation, we found an increase in OSI and vice versa. For both the ideal and patient-specific aneurysm geometries investigated in this work, we observed that the degradation-related enhancement of this inverse correlation between TAWSS and OSI occurs predominantly at low-WSS regions.

Although intra-aneurysm flow patterns and hemodynamic quantities depend on the specific geometry (Varble et al., 2016; Wang et al., 2020, 2021a; Khan et al., 2021), effects of tissue degradation turn out to be qualitatively similar in all the investigated cases, i.e., for different geometries, degradation intensities, heart rates and blood pressures: at low-WSS sites, degradation leads to a lower TAWSS and at the same time a higher OSI.

Low TAWSS accompanied by high OSI has been associated with the thrombus accumulation in aneurysms (Frösen et al., 2012; Zambrano et al., 2016; Kelsey et al., 2017; Ong et al., 2019) via promoting a prothrombogenic phenotype (Chiu and Chien, 2011; Wolberg et al., 2012), and with the aneurysm's progression and a rupture-prone phenotype (Les et al., 2010; Xiang et al., 2011; Meng et al., 2014; Zhang et al., 2016; Liu et al., 2019a). Such hemodynamic characteristics also promote the formation of atherosclerotic plaques on aneurysms (Galis et al., 1999; Tateshima et al., 2008; Chiu and Chien, 2011; Yang et al., 2011; Frösen et al., 2012) and thus make the aneurysmal walls vulnerable to rupture. A number of molecules are involved in the formation and rupture of aneurysms. The combination of low WSS and high OSI is known to elicit the inflammatory response in the endothelium (Meng et al., 2014). When endothelial cells are exposed to low and oscillatory shear stress, they respond by producing reactive oxygen species (Galis et al., 1999; Chiu and Chien, 2011), recruiting more inflammatory cells (van Varik et al., 2012), upregulating vascular cell adhesion molecules and cytokines in the vessel wall (Aoki et al., 2011; van Varik et al., 2012), and increasing endothelial permeability (Malek et al., 1999; Bian et al., 2009; Chiu and Chien, 2011). Conway et al. (2010) have reported that low average shear stress and high OSI (or flow reversal) act as significant mechanical stimuli on the regulation of endothelial cell gene expression and monocyte adhesion, respectively. A “sticky” and “leaky” proinflammatory endothelium tends to promote leukocyte transmigration into the vascular wall during the progression of aneurysms (Chiu and Chien, 2011; Meng et al., 2014). Low WSS together with high OSI also causes smooth muscle cells to undergo phenotypic modulation and apoptosis (Hsu et al., 2011; Liu et al., 2019b), and facilitates inflammatory cell infiltration (Malek et al., 1999; Nixon et al., 2010; Soldozy et al., 2019). These inflammatory responses in vessel walls can produce matrix metalloproteinases to destroy internal elastic lamina and degrade the extracellular matrix (Galis et al., 1994), thereby leading to an imbalance between the constructive and destructive processes (Meng et al., 2014).

Importantly, if we combine our findings with prior observations, we obtain a ‘degradation loop': Degradation of an aneurysm wall leads to lower TAWSS and higher OSI which correspond to a favorable environment for pathological changes such as atherosclerosis and thrombus formation in the aneurysm; such flow environment, in turn, may induce further degradation. Hence, the present study suggests that degradation enhances this pathological process via a self-amplification mechanism: sites with a low TAWSS and at the same time a high OSI experience still lower TAWSS and higher OSI during degradation.

A degradation process may thus self-amplify/accelerate itself via enhancing the contrast between TAWSS and OSI. That is, tissue degradation may promote the aneurysm's pathological progression to rupture by accelerating the usually slow processes, which leads to enhanced vulnerability of the wall.

Several improvements can be added to the numerical approach used in this study. First, it assumes the blood to be a Newtonian fluid with constant viscosity. This is a good approximation at low flow rates but may need correction in large arteries, where shear rate varies over a wider range from the center toward the vessel wall. Second, active response and fatigue of the aneurysmal wall are not accounted for in the material model. Third, idealized fiber orientations are employed. Fourth, a uniform wall thickness is assumed in this study. Fifth, we assume a parabolic velocity profile at the inlet. The main reason for these simplifying assumptions is that the patient-specific data of the mechanical response of aneurysmal walls, the fiber architecture, the distribution of wall thickness and the boundary conditions are not yet available. This is presumably related to the complexity of the degradation phenomenon and difficulties associated with the measurements of these properties of the arterial wall during the degradation process. Indeed, the model can be easily improved if the corresponding experimental and clinical data become available.

This study is a first step to investigate the effects of tissue degradation on the hemodynamic factors and the potential evolution of an aneurysm. In the future, if clinical/experimental data of fiber orientations in an individual aneurysm becomes available, the current model could be used to predict if the aneurysm is susceptible to develop further damage and, if so, to estimate the regions where rupture is more likely to occur.

## 5. Conclusion

Using computer simulations with an idealized aneurysm and two patient-specific ones, we investigated the effects of tissue degradation on the mechanical stimuli, time-averaged wall shear stress and oscillatory shear index, which are commonly associated with aneurysms.

We found that the degradation-induced variations of TAWSS and OSI are opposite to each other: If in the course of degradation the time-averaged wall shear stress is decreased at a given site, we observed an increase in OSI at the same site and vice versa. This process is most prominent at sites that are already subject to a low TAWSS and at the same time a high OSI. This finding turned out to be robust and was confirmed in the case of all the three investigated aneurysm geometries and different degradation intensities, heart rates, and blood pressures.

Our findings are discussed in the light of the present state of understanding in the literature regarding biomechanical mechanisms responsible for aneurysm development and rupture. We argue that degradation is likely to enhance the existing contrast between these two hemodynamic factors (in particular, at sites subject to a low time-averaged wall shear stress and a high oscillatory shear index), and thereby pave the way for the next degradation step. This study thus underlines the importance of the inverse correlation between TAWSS and OSI as an independent risk factor for aneurysm degradation and rupture. Further investigations are warranted to examine these findings in a clinical setting.

## Data Availability Statement

The original contributions presented in the study are included in the article/Supplementary Material, further inquiries can be directed to the corresponding author/s.

## Author Contributions

HW and FV designed the details of the specific computational and analysis protocols including the study of patient-specific geometries. HW, VV, KU, and DB contributed to the algorithmic implementation. HW performed the simulations and analyses and wrote the first draft. HW and FV revised the manuscript. All authors contributed equally to the development of the original hypothesis of self-amplification and the design of suitable numerical analysis, read, and approved the final version of the manuscript.

## Funding

This work was performed with support from the IMPRS-SurMat program and by the German Research Foundation (DFG) within SFB-TRR 287/1 (project A4).

## Conflict of Interest

The authors declare that the research was conducted in the absence of any commercial or financial relationships that could be construed as a potential conflict of interest.

## Publisher's Note

All claims expressed in this article are solely those of the authors and do not necessarily represent those of their affiliated organizations, or those of the publisher, the editors and the reviewers. Any product that may be evaluated in this article, or claim that may be made by its manufacturer, is not guaranteed or endorsed by the publisher.
