# Supplementary material for: On the Potential Self-Amplification of Aneurysms Due to Tissue Degradation and Blood Flow Revealed From FSI Simulations
Source: Front Physiol. 2021 Dec 10;12:785780. doi: 10.3389/fphys.2021.785780 (PMC8709128; doi:10.3389/fphys.2021.785780)
Supplement: Supplementary file 1 [file Presentation_1.pdf]

Supplementary Material

TISSUE DEGRADATION MODEL

The tissue degradation model used in this work has been developed by Daniel Balzani and coworkers (Balzani et al., 2012; Anttila et al., 2019). In a previous study, we have used this model to study the interaction between tissue degradation and blood flow (Wang et al., 2021). Here we give a brief outline of this method.

The total strain energy function of the tissue degradation model is chosen to be (Balzani et al., 2012)

$$\Psi^{\rm tot} = \Psi^{\rm iso} + \Psi^{\rm vol} + \sum_{i=1}^{2} \Psi_i^{\rm ti}. \tag{S1}$$

Here, $\Psi^{\rm iso} = c_1(I_1/I_3^{1/3} - 3)$ describing the ground matrix material (also known as elastin) as incompressible Neo-Hookean; $\Psi^{\rm vol} = \epsilon_1(I_3^{\epsilon_2} + 1/I_3^{\epsilon_2} - 2)$ (Hartmann and Neff, 2003) serving as volumetric penalty function to account for near-incompressibility. $I_1 = {\rm tr}\boldsymbol{C}$ and $I_3 = \det\boldsymbol{C}$ denote the first and the third invariants of the right Cauchy-Green tensor $\boldsymbol{C} = \boldsymbol{F}^{\rm T}\boldsymbol{F}$, while $\boldsymbol{F}$ is the deformation gradient tensor. The effective elasticity tensor is computed via $\mathbb{C} = 4\partial^2_{\boldsymbol{CC}}\Psi$ (Balzani et al., 2012).

The effect of damage is accounted for across two phenomenological fiber families $i = 1, 2$ as

$$\Psi_i^{\rm ti} = \alpha_1 \langle (1 - D_i)[\kappa I_1 + (1 - 1.5\kappa)K_3^i] - 2\rangle^{\alpha_2}, \tag{S2}$$

describing the material behavior of collagen fibers. D_i are scalar damage functions for each fiber family i used to capture remnant strains (i.e., strain at zero stress level after unloading) within the fibers and the stress-softening effect. The Macaulay brackets, $\langle(\cdot)\rangle = [(\cdot) + |(\cdot)|]/2$, filter out positive values. $K_3^i = {\rm tr}[{\rm cof}\boldsymbol{C}(\boldsymbol{1} - \boldsymbol{M_i})]$ is the fundamental polyconvex function (Schröder and Neff, 2003) with the definition of the structural tensor $\boldsymbol{M_i} = \boldsymbol{A_i} \otimes \boldsymbol{A_i}$, given in terms of each fiber direction vector $\boldsymbol{A}_i$. The cofactor is defined as ${\rm cof}\boldsymbol{C} = \det\boldsymbol{C}\boldsymbol{C}^{-1}$. Here, $c_1,\ \epsilon_1,\ \epsilon_2,\ \alpha_1, \alpha_2$ and κ are material parameters.

The damage function D_i for a fiber family i is defined as

$$D_i(\beta_i) = D_{{\rm s},i}\left[1 - \exp\left(\frac{\ln(1 - r_{\rm s})}{\beta_{\rm s}}\beta_i\right)\right], \tag{S3}$$

where the maximally reachable damage value for fixated load levels is denoted by $D_{{\rm s},i} \in [0, 1)$, the fraction of the maximum damage is $r_{\rm s} = 0.99$, and $\beta_{\rm s} > 0$ is the value of the internal variable β_i corresponding to damage saturation. The internal variable is defined as $\beta_i = \langle\tilde{\beta}_i - \tilde{\beta}_i^{\rm ini}\rangle$, with $\tilde{\beta}_i = \int_0^t \langle\dot{\Psi}_i^{{\rm ti},0}(s)\rangle {\rm d}s$ allowing for continuous damage evolution for loading and re-loading paths and $\tilde{\beta}_i^{\rm ini}$ denoting the damage initiation threshold. t indicates the time at the current state, and $\dot{\Psi}_i^{{\rm ti},0}$ is the first time derivative of the fictitiously undamaged (effective) strain energy density $\Psi_i^{\rm ti,0}$ in fiber direction i. The maximally reachable damage value for fixated load levels $D_{{\rm s},i}$ is expressed as

$$D_{{\rm s},i}(\gamma) = D_\infty\left[1 - \exp\left(\frac{\ln(1 - r_\infty)}{\gamma_\infty}\gamma_i\right)\right], \tag{S4}$$

with the predefined converging limit for the overall damage value $D_\infty \in [0, 1)$ and $\gamma_\infty > 0$ representing the value of the internal variable γ_i reached at the limit fraction $r_\infty = 0.99$.

In order to ensure that $D_{\mathrm{s},\,i}$ remains unchanged during a cyclic process under fixed maximum load levels, the second internal variable

$$\gamma_i = \max_{s\in[0,t]} \langle \Psi_i^{\mathrm{ti},\,0}(s) - \Psi_{\mathrm{ini},\,i}^{\mathrm{ti},\,0} \rangle \tag{S5}$$

is defined as the maximum value of the effective energy reached up to the current state at t. $\Psi_{\mathrm{ini},\,i}^{\mathrm{ti},\,0}$ denotes the effective strain energy density at an initial damage state obtained at the limit of the physiological domain. The damage saturation criterion is expressed as

$$\phi_i := \langle \Psi_i^{\mathrm{ti},\,0}(s) - \Psi_{\mathrm{ini},\,i}^{\mathrm{ti},\,0} \rangle - \gamma_i \leq 0. \tag{S6}$$

In this model, different degradation intensities can be simulated by adapting the damage parameter γ_∞. A small degree of damage can be simulated by increasing the value of γ_∞ (Wang et al., 2021). Compared with $\gamma_\infty = 11$ kPa, we use $\gamma_\infty = 18$ kPa to simulate a smaller degradation (which may represent the accumulation (overall effect) of damage for a shorter time).

REFERENCES

Anttila, E., Balzani, D., Desyatova, A., Deegan, P., MacTaggart, J., and Kamenskiy, A. (2019). Mechanical damage characterization in human femoropopliteal arteries of different ages. *Acta Biomaterialia.* 90, 225–240

Balzani, D., Brinkhues, S., and Holzapfel, G. (2012). Constitutive framework for the modeling of damage in collagenous soft tissues with application to arterial walls. *Comput. Methods Appl. Mech. Engrg.* 213-216, 139–151

Hartmann, S. and Neff, P. (2003). Existence theory for a modified polyconvex hyperelastic relation of generalized polynomial-type in the case of nearly-incompressibility. *Int J. Solids Struct* 40, 2767–2791

Schröder, J. and Neff, P. (2003). Invariant formulation of hyperelastic transverse isotropy based on polyconvex free energy functions. *Int J. Solids Struct* 40, 401–445

Wang, H., Uhlmann, K., Vedula, V., Balzani, D., and Varnik, F. (2021). Fluid-structure interaction simulation of tissue degradation and its effects on intra-aneurysm hemodynamics. *bioRxiv* doi:https://doi.org/10.1101/2021.09.01.458529
